# Placental mitochondria in high-altitude pregnancy: metabolic adaptations and a toolkit for respiratory assessment

**DOI:** 10.1098/rstb.2024.0175

**Published:** 2025-08-21

**Authors:** Andrew Murray, Jenna Armstrong, Katie O'Brien

**Affiliations:** ^1^ Department of Physiology, Development and Neuroscience, University of Cambridge, Cambridge CB2 3EL, UK; ^2^ Department of Biomedical Informatics, University of Colorado Anschutz Medical Campus, Aurora, CO 80045, USA

**Keywords:** placenta, mitochondria, pregnancy, high altitude, metabolism

## Abstract

Ascent to high altitude is accompanied by physiological responses that, to an extent, mitigate the challenge of hypobaric hypoxia, maintaining arterial oxygen content and convective oxygen delivery. Nevertheless, arterial oxygen tension (*p*O_2_) remains low and tissue hypoxia persists, posing a challenge for metabolic and redox homeostasis, and therefore function. The physiological challenge of life at high altitude is well exemplified by human pregnancy. Infant birthweight falls at high altitude, and there is a greater incidence of pregnancy complications, such as preeclampsia and fetal growth restriction; however, relative protection is seen in Tibetan and Andean populations. There is evidence to implicate a role for placental metabolic reprogramming and mitochondrial alterations in this context, while metabolic adaptations likely contribute to an integrated response that supports healthy pregnancies in highlander populations at altitude. Here, we outline the evidence to support a role for placental mitochondria in healthy and pathological pregnancies at high altitude. We propose that a better understanding of placental metabolic responses to tissue hypoxia would have important implications both for pregnancies at high altitude and in complicated pregnancies more generally, and we outline technical developments that allow the comprehensive assessment of placental mitochondrial respiration in an integrated, physiologically relevant context.

This article is part of the discussion meeting issue ‘Pregnancy at high altitude: the challenge of hypoxia’.

## 1. Introduction

With ascent to high altitude, barometric pressure falls decreasing the partial pressure of inspired O_2_ (*p*_i_O_2_). This impedes oxygen delivery to the tissues, giving rise to tissue hypoxia and the accompanying challenge of maintaining cellular metabolic and redox homeostasis. Physiological responses to hypoxic challenge include those that mitigate the fall in convective oxygen delivery through, e.g. increased ventilation, cardiac output, angiogenesis and oxygen carriage capacity (i.e. red blood cell mass) [1]. This increase in haematocrit offsets the fall in haemoglobin O_2_ saturation at altitude, effectively maintaining arterial oxygen content (*C*_a_O_2_) even up to 7000 m [2]. However, arterial oxygen pressure (*p*O_2_) remains low, limiting oxygen diffusion and necessitating metabolic responses that alter cellular oxygen demand [3]. Despite these adaptive responses, tissue hypoxia persists and is thus a constant experience for the estimated 80 million people inhabiting regions over 2500 m above sea level, as well as those who visit these regions each year.

The physiological challenge of life at high altitude is well exemplified by human pregnancy. Infant birthweight falls by approximately 100 g for each 1000 m increment in elevation [4], and there is an increased incidence of pregnancy complications, such as preeclampsia and gestational hypertension [5]. Alterations in placental metabolism have been implicated in the mechanisms that result in altitude-dependent fetal growth restriction, and as the end-consumers of oxygen, placental mitochondria are of particular interest [6]. There remains a need to better understand the impact of hypobaric hypoxia on placental mitochondrial respiration, including substrate metabolism, electron transfer system (ETS) capacity and oxidative phosphorylation (OXPHOS) coupling efficiency. Moreover, studies of placental mitochondria from native highlander populations could reveal adaptive bioenergetic strategies that offset the metabolic and redox challenge of hypoxia.

Human populations have inhabited high-altitude regions on the Tibetan Plateau, the Andean Altiplano and the Highlands of Ethiopia for many thousands of years. This has allowed the selection of physiological traits, underpinned by genetic factors, that support their capacity to live, to work and crucially to reproduce at high altitude [7]. These include many strong signals of selection around genes that regulate metabolism and associated traits [8]. Of note, birthweight at high altitude is relatively protected in both Andean and Tibetan highlanders compared with lowlanders [9–11], and while incidence of preeclampsia remains high during pregnancy, there is again relative protection in comparison with women of lowlander heritage [5,11]. The mechanisms that potentially explain this protection include enhanced uterine artery blood flow [12] and elevated maternal antioxidant capacity [13], both of which have been reported in Andean pregnancies. These features are likely to be the components of an integrated response that includes the protection of placental metabolism and function, and therefore fetal growth.

## 2. Placental metabolism and mitochondria

The human placenta has a significant demand for ATP to support energetic requirements, which include protein synthesis (e.g. for growth and hormone synthesis) and transport functions [14]. In the term placenta, two morphologically distinct subpopulations of mitochondria have been identified, with proteomic and respirometric analysis of mitochondrial isolates suggesting functional differences *in vivo* [15,16]. At the syncytiotrophoblast, a smaller, more fragmented population of mitochondria is characterized by a relatively low capacity for OXPHOS but a greater capacity for steroid synthesis, whereas in the cytotrophoblast a more reticular mitochondrial population exhibits greater oxidative capacity [16].

Glucose is the major metabolic substrate that meets placental energetic demand [17], supporting ATP generation either via glycolysis or through mitochondrial OXPHOS via pyruvate. Glucose oxidation results in a higher net ATP yield than glycolysis (*ca* 30 moles ATP per mole glucose, compared with the net production of 2 moles of ATP per mole of glucose via glycolysis) but consumes O_2_ in the process. In the relatively low-oxygen environment of early pregnancy (*p*O_2_ ≈ 20 mmHg) glycolysis therefore dominates and is sustained through the reoxygenation of pyridine nucleotides by pathways of sorbitol synthesis [18]. This seemingly inefficient use of substrate conserves carbon skeletons for biosynthesis, and can be sustainable pending a constant supply of glucose. Towards the end of the first trimester, however, conversion of the maternal spiral arteries results in a rapid increase in uterine artery blood flow and an elevation in placental *p*O_2_ from around 20 mmHg towards 55 mmHg [19,20], allowing a greater yield of ATP via OXPHOS.

In comparison with glucose metabolism, fatty acid oxidation (FAO) is considered to make a relatively minor contribution to placental ATP demands; however, the human placenta expresses enzymes for β-oxidation of fatty acids, while trophoblast cells in culture can oxidize fatty acids [21,22]. FAO may be important earlier in pregnancy, with an inverse relationship seen between enzyme activity and maternal gestational age across the second and third trimesters [21]. In comparison with glucose oxidation, FAO results in a higher yield of ATP per carbon unit, but more oxygen is consumed in the process. Owing to the compartmentation of β-oxidation, fatty acid metabolism is also critically dependent on mitochondrial capacity.

The placenta is thus both a consumer of substrates and oxygen as well as a conduit of nutrients and oxygen to the fetus, and therefore it is vital that placental metabolism is modulated to balance its own metabolic demands against that of the fetus. Central to this modulation are the placental mitochondria. Measurements of glucose utilization by the term placenta made *in vivo* are in the region of 50 µmol min^−1^ (kg tissue)^−1^, exceeding that of the fetal tissues (*ca* 30 µmol min^−1^ (kg tissue)^−1^) [23]. Fetal glucose utilization correlates with birthweight [23], underlining the importance of maintaining fetal glycaemia to protect growth. Correspondingly, in fetal growth restriction, the maternal–fetal glucose concentration gradient is wider than in healthy pregnancies, and corresponds to clinical severity [24]. Intriguingly, fetal glucose consumption does not correlate with uteroplacental glucose uptake, which instead appears to determine the rate of placental glucose consumption [23]. This highlights a role for the modulation of placental metabolism to ensure optimal fetal glucose and oxygen availability, with the relative balance of glycolysis, glucose oxidation and FAO at the placenta effectively buffering fetal nutrient and oxygen availability against fluctuations in maternal supply.

## 3. Placental mitochondria in high-altitude pregnancy

In high-altitude pregnancy, maternal *C*_a_O_2_ is typically maintained through erythropoiesis [25,26], with the importance of this seen through correlations of lower maternal *C*_a_O_2_ with lower birthweight [27]. In Tibetans, reproductive success (i.e. number of live births) was found to correspond to a maternal capacity to maintain *C*_a_O_2_ through an appropriately moderate haematocrit (maintaining O_2_ carriage capacity, but avoiding excess viscosity) alongside a high percentage of haemoglobin–O_2_ saturation [28]. In human pregnancy, uteroplacental blood flow is high and, as such, convective oxygen delivery would appear to exceed placental and fetal oxygen demand; however, this serves todecrease the *p*O_2_ gradient between the maternal artery and uterine vein [29]. As uterine venous *p*O_2_ sets the upper limit for diffusion, the high uteroplacental blood flow coupled with *C*_a_O_2_ likely serves to maintain oxygen delivery to the placenta and fetus [29,30]. Uterine artery blood flow is elevated in Tibetans and Andeans at high altitude in comparison with native lowlanders [29], and the resulting defence of uterine venous *p*O_2_ (supported by estimates in Andean pregnancies) might explain the relative protection of birthweight in highlander populations [12,30].

In pregnancies of European and Andean woman at 3600 m, however, fetal oxygen consumption (at least, when reported per unit of tissue mass) was found to be similar to that seen in sea-level pregnancy, despite a lower fetal blood flow, with protection arising from increased fetal haematocrit and enhanced fetal oxygen extraction [31]. The greater fetal haematocrit at high altitude was accompanied by elevated levels of erythropoietin (EPO) in the fetal circulation in all pregnancies, although, notably, fetal EPO levels were higher in Europeans at 3600 m than in Andeans at the same altitude [31]. Fetal EPO has been proposed to be a marker of fetal hypoxia [32,33], and thus this finding might indicate that adaptations upstream of the fetal circulation (e.g. uterine artery blood flow, placental metabolism or transplacental O_2_ transfer) serve to minimize the degree of hypoxia experienced by the fetus in Andean highlander pregnancy in comparison with that of Europeans. Strikingly, umbilical venous oxygen content at 3600 m was maintained or perhaps even elevated in high-altitude pregnancies compared with sea-level pregnancy, despite a lower *p*O_2_ [31]. Instead, a finding of fetal hypoglycaemia (2.9 mM compared with 3.5 mM at sea level) and consequently lower fetal glucose consumption was postulated to constrain fetal growth [34]. As such, high-altitude pregnancy would appear to represent a paradigm in which placental metabolic modulation through a glycolytic switch serves to protect fetal oxygen content by limiting OXPHOS, but at the cost of enhanced placental glucose demand and fetal hypoglycaemia [6,34].

In support of this concept, metabolomic analysis of placentas from pregnancies at 3100 m showed lower glucose concentrations and elevated lactate, suggestive of a metabolic switch to enhanced glycolysis, as well as resistance to oxidative stress during labour [35], all of which may indicate a lower mitochondrial content. Moreover, placental oxidative capacity correlated negatively with umbilical venous *p*O_2_ in healthy, normotensive Andean pregnancies at altitude, suggesting that the suppression of placental oxidative metabolism could protect oxygen delivery to the fetal circulation [36]. This presumed adaptive suppression of mitochondrial respiratory capacity was associated with carriage of a genetic region under selective pressure within *PTPRD*, encoding protein tyrosine phosphatase receptor-δ [36]. The mechanisms linking *PTPRD* and placental respiratory capacity in Andeans are not understood, but modulation of the mitochondrial respiratory chain [37,38] and insulin receptor signalling [39] are potential avenues for future investigation.

Metabolic responses to hypoxia are mediated at least in part by the hypoxia-inducible factor (HIF) pathway [40]. In most tissues, under conditions of normoxia, the HIF-1α and HIF-2α isoforms are expressed and transcribed, but hydroxylated in an oxygen-dependent fashion by the prolyl-hydroxylase (PHD) enzymes [40]. Thus, HIF-1α and HIF-2α are tagged for ubiquitination by the von Hippel–Lindau (VHL) protein, and degraded under the action of the proteasome [40]. Under hypoxic conditions, however, HIF-1α and HIF-2α are stabilized and form heterodimers with HIF-1β [40]. These heterodimers bind to hypoxia response elements in the promoter regions of hundreds (if not thousands) of target genes encoding factors that underpin the systemic and cellular responses to hypoxia [38]. This includes many genes encoding enzymes of the glycolytic pathway [41]. HIF activation thereby enhances glycolysis but can also modulate other aspects of metabolism, for example inhibiting pyruvate oxidation and increasing lactate production [42,43] and impairing mitochondrial electron transfer [43]. Moreover, HIF activation has been associated with mitophagy [44], resulting in loss of mitochondrial content in some tissues [45], which may limit the capacity for mitochondrial reactive oxygen species (mtROS) generation and oxidative stress under hypoxic conditions [46]. Indeed, interactions between ROS and the PHD enzymes might modulate the HIF response [47], implicating an oxygen-sensing role for the mitochondria [48].

Analysis of mitochondrial ETS proteins in placentas from pregnancies at 3100 m revealed lower levels of representative subunits of complexes I–IV in comparison with sea-level placentas [49]. Of note, this was not accompanied by differences at the gene expression level, suggesting a post-transcriptional mechanism of regulation [49]. A strong candidate for this regulation is the microRNA mir210, which is upregulated downstream of HIF1 activation [50] and was found to be elevated in high-altitude placentas [49]. This was in turn associated with decreased expression of the mir210 targets: *ISCU1/2* encoding the iron–sulfur cluster assembly enzyme and *COX10* encoding an assembly factor for cytochrome *c* oxidase [49], both of which maintain ETS capacity.

Several studies in placental explants or trophoblast-like cell lines have supported a role for hypoxia-led suppression of mitochondrial respiratory capacity. Respiration supported by substrates for complexes I and IV was suppressed in hypoxic placental fibroblasts and JEG3 cells in comparison with normoxic cells, and this was accompanied by lower levels of corresponding ETC complex proteins as well as COX10 and ISCU1/2, and increased mir210 in hypoxic fibroblasts [49]. In BeWo cells, hypoxic exposure resulted in suppression of respiratory capacity and ETC complex proteins in one study [49], and decreased markers of mitochondrial content and biogenesis in another study alongside increased expression of glycolytic genes, with some of these findings also seen in placental explants exposed to hypoxia [51]. In HTR-8/SVneo cells treated with cobalt chloride (CoCl_2_) to induce HIF-1α stabilization, decreased ATP levels and a diminished mitochondrial proton gradient were seen, alongside decreased expression of ETC components and increased markers of mitophagy [52]. Similar changes in the expression of ETC proteins were seen alongside HIF-1α stabilization in placentas from pregnancies diagnosed with fetal growth restriction [52].

Further support for metabolic reprogramming in the hypoxic placenta arises from studies of preeclampsia in human pregnancy. Preeclampsia is characterized by impaired uteroplacental blood flow and placental oxidative stress [53,54]. While the enhanced oxidative stress and hypoxia arising in preeclampsia might impact on placental mitochondrial function [55,56], in one case study, familial mitochondrial abnormalities were associated with recurrent preeclamptic pathologies [57], suggesting that defective mitochondrial respiratory function might also play a causative role, as well as being a downstream consequence of preeclampsia. Notably, mir210 was upregulated in placentas from late-onset preeclampsia alongside decreased expression of *ISCU1/2* [58]. While there are reports of lower mitochondrial content and increased markers of glycolysis in placentas from preeclamptic pregnancies [59], other reports have noted differential changes in pre-term preeclamptic placentas compared with those that progress to term delivery, with greater mitochondrial respiratory capacity in those that progress to term compared with normotensive controls [60]. This possibly indicates a role for an adaptive mitochondrial response to the pathology, perhaps accompanied by a lower burden of endoplasmic reticulum stress [61]. Of note, severe, early-onset preeclampsia is associated with placental mitochondrial swelling and suppressed respiration, alongside activation of the mitochondrial unfolded protein response and markers of endoplasmic reticulum stress [62].

Incidence of preeclampsia is elevated at high altitude [5], and is associated with maternal and fetal hypoxia [30,63], as well as suppressed placental mitochondrial function in comparison with normotensive pregnancies at the same altitude [36]. In sheep, chronic hypoxia during pregnancy mimics many aspects of the aetiology of preeclampsia [64], including suppressed mitochondrial respiration [65,66]. Moreover, the relationship between placental oxidative metabolism and umbilical venous *p*O_2_ seen in normotensive pregnancies at altitude was lost in preeclamptic pregnancies, suggesting a dysregulation of placental mitochondrial respiration [36].

A further pathway downregulated in many oxidative tissues in response to hypoxia is FAO, with HIF activation decreasing FAO capacity by suppressing the fatty acid-activated transcription factor peroxisome proliferator-activated receptor α (PPARα) in some tissues [67–70], and suppression of FAO seen in some highlander populations [8,71]. This may serve to enhance the oxygen efficiency of ATP synthesis, but at the possible cost of accumulating lipotoxic lipid species at altitude, at least in lowlanders, with Sherpas for instance showing protection [71]. Placental expression of FAO genes is downregulated in preeclampsia [72], and FAO capacity is suppressed in response to oxidative stress in term placentas [73]. Moreover, protection of placental mitochondrial respiration in normotensive versus preeclamptic pregnancies at high altitude was associated with the selection of a genetic region encompassing the gene *CPT2*, encoding the protein carnitine palmitoyl-transferase 2, a key component of mitochondrial fatty acid import [36,74]. This further implicates a role for the regulation of placental FAO under conditions of hypoxia and/or oxidative stress, though the mechanistic basis for this protection is yet to be resolved. Moreover, the impact of high-altitude hypoxia during pregnancy on placental FAO is unknown, although studies in mice indicate a suppression of placental FAO with exposure to chronic hypoxia during pregnancy [75].

Taken together, there is a wealth of evidence to support a role for mitochondrial modulation, including ETS capacity and substrate metabolism in supporting placental function and fetal growth and development at high altitude. At present, alterations in placental mitochondrial respiration in response to hypoxia have largely been inferred from proxy analysis of gene/protein expression or spectrophotometric analysis of enzyme activities [49,52,59], and there remains a need to understand mitochondrial respiration in a more integrated, physiologically relevant system. Moreover, there are a striking paucity of data concerning placental FAO in human pregnancy at high altitude.

## 4. A toolkit for the assessment of placental mitochondrial respiration in high-altitude pregnancy

High-resolution respirometry is a gold standard technique for the measurement of O_2_ flux in biological samples *ex vivo*, allowing the assessment of mitochondrial respiration under the control of different substrate-led pathways and in different respiratory states [76]. Measurement of O_2_ consumption, which is coupled to electron flow, can be made in intact cells and tissue preparations, but in this case the experimental manipulation of respiration is limited to the use of cell-membrane-permeable substrates, inhibitors and uncoupling agents, limiting the degree to which the mitochondrial phenotype can be elucidated. A greater degree of experimental control can be achieved by removal of the cell membrane barrier function via mitochondrial isolation, selective cell membrane permeabilization or preparation of tissue homogenates. In each case, preparation protocols should be optimized to ensure intact mitochondrial membranes are preserved, retaining the bioenergetic coupling of substrate oxidation (i.e. electron flow to O_2_, supporting proton pumping) and ADP phosphorylation to ATP at the F_1_F_o_–ATP synthase, supported by proton influx. Quality control steps integrated within respirometry assays can be used to assess the mitochondrial membrane integrity. Outer mitochondrial membrane intactness can be assessed through the administration of exogenous reduced cytochrome *c*, which should result in no significant increase in oxygen consumption, indicating that an intact outer membrane has effectively retained endogenous cytochrome *c*. Inner mitochondrial membrane integrity can be observed through an increase in O_2_ consumption upon the addition of exogenous ADP, which stimulates proton influx via ATP synthase, thereby allowing more proton pumping and electron flow to O_2_.

Mitochondrial membrane integrity is typically disrupted by freeze–thawing, and so respirometry is traditionally carried out on fresh cell or tissue preparations. Recent technical developments have suggested that measurements of respiratory capacity can be made using preparations of previously frozen tissue through the addition of cytochrome c to reconstitute the ETS and selection of appropriate substrates (e.g. NADH for direct electron transfer to complex I) [77]. While this approach allows the use of archived tissue, the information that can be gleaned from such preparations is limited by the uncoupled nature of freeze–thawed mitochondria (and absence of a proton gradient) and the loss of mitochondrial matrix enzymes, preventing assessment of FAO, for example. Respirometry using fresh tissue therefore allows the comprehensive analysis of mitochondrial respiratory function under the control of substrate-led pathways and the inner membrane proton gradient—an integrated, physiologically relevant bioenergetic state.

The limitations of freeze–thawed preparations, and thus the desirability of using fresh tissue, do, however, bring significant technical challenges. For instance, when seeking to measure respiration in placental samples post-delivery, the unpredictable timing of sample availability would require a calibrated oxygraph and trained operator to be on standby in close proximity to the delivery unit for several hours or longer before time-consuming assays can take place. Moreover, technical challenges can be further exacerbated when conducting respirometry in remote locations at high altitude, requiring significant logistical support [78].

**Figure 1.**
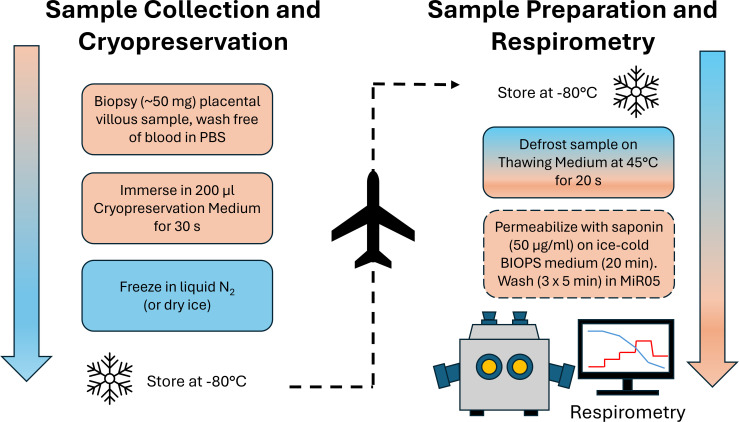
Schematic workflow for the cryopreservation and storage of placental samples, and the subsequent preparation for high-resolution respirometry. For the composition of media, see main text. PBS, phosphate-buffered saline; BIOPS, biopsy preservation solution; MiR05, mitochondrial respiration medium.

To circumvent these limitations, we developed and optimized a straightforward technique that allows the preservation of intact mitochondria in frozen preparations of human placenta (figure 1) [79]. Placental biopsies are obtained and processed as per established protocols, essentially as described in [80]. The basal plate is first removed before samples of exposed villous tissue (*ca* 50 mg) are collected at randomly selected sampling sites approximately midway between the placenta margin and cord insertion. Samples are washed free of blood in ice-cold phosphate-buffered saline (PBS) before being immersed in 200 µl of a cryopreservation solution (0.21 M mannitol, 0.07 M sucrose, 0.02 M HEPES, 30% dimethyl sulfoxide (DMSO), 70% double-distilled H_2_O, pH 7.5) for 30 s, before rapidly freezing in liquid N_2_ (or by completely covering the sample tube in dry ice). Samples can then be maintained at −80°C for several months [79], and shipped frozen on dry ice. To date, we have collaborated with research groups in the United Kingdom, United States, Canada and Bolivia, collecting and cryopreserving more than 300 term placental samples at different altitudes. As seen through within-assay assessment of mitochondrial membrane integrities, cryopreservation and recovery of intact mitochondria have been effective in >95% of samples. Results from some of these studies, including our work on placental samples from healthy and preeclamptic pregnancies at high altitude, have been discussed in earlier sections of this article [36].

Prior to conducting respirometry, cryopreserved samples are thawed in pre-warmed (45°C) thawing medium (0.25 M sucrose, 0.1 M Tris–HCl, pH 7.5) in a 4:1 ratio of medium volume to frozen sample volume, and placed in a water bath at 45°C for 20 s. Thawed samples are immediately transferred to ice-cold biopsy preservation medium (BIOPS; 2.77 mM CaK_2_EGTA, 7.23 mM K_2_EGTA, 6.56 mM MgCl_2_·6H_2_O, 20 mM taurine, 15 mM sodium phosphocreatine, 20 mM imidazole, 0.5 mM dithiothreitol (DTT), 50 mM K-2-(*N*-morpholino)ethanesulfonic acid (MES), 5.77 mM Na_2_ATP, pH 7.1) and gently teased apart using fine, non-magnetic forceps. Selective cell membrane permeabilization is carried out through the addition of saponin (50 µg ml^−1^) with gentle rocking on ice for 20 min. Samples are then washed for 5 min × 3 in ice-cold MiR05 medium (0.5 mM EGTA, 3 mM MgCl_2_·6H_2_O, 60 mM K-lactobionate, 20 mM taurine, 10 mM KH_2_PO_4_, 20 mM HEPES, 110 mM sucrose, 1 g l^−1^ defatted bovine serum albumin, pH 7.1) before 25−40 mg of permeabilized tissue is transferred to each oxygraph chamber with 2 ml MiR05 at 37°C.

Thus, respiration can be measured in the absence of ADP (LEAK state), in the presence of ADP to stimulate OXPHOS state, and in the presence of inner mitochondrial membrane uncouplers to reveal electron transfer capacity (ET state) [81]. Each of these states, in turn, can be measured in the presence of different electron donors (i.e. substrates), administered alone or in combination, e.g. fatty acid-derived substrates (acyl-CoA or acyl-carnitines), electron donors for complex I-supported respiration (e.g. pyruvate + malate or glutamate + malate), complex II-supported respiration (succinate), complex III-supported respiration (duroquinol) or complex IV-supported respiration (TMPD + ascorbate). The inclusion of specific inhibitors of pathway components allows greater experimental flexibility and further insight into ETS control. The flexibility of this approach allows the design of bespoke assays to address specific scientific questions. Here, we highlight two sample protocols that we have used in placental preparations for (i) the assessment of respiratory capacity supported by ETS components (table 1) and (ii) the assessment of FAO and its contribution to ETS capacity (table 2). In both assays, outer mitochondrial membrane intactness is assessed through the addition of exogenous cytochrome *c*, as detailed earlier.

**Table 1.** Example substrate/uncoupler/inhibitor titration protocol (with final concentrations) to probe mitochondrial respiration supported by electron transport system (ETS) components in saponin-permeabilized placental samples. ADP = adenosine diphosphate; ET = electron transfer; FCCP = carbonyl cyanide p-(trifluoromethoxy) phenylhydrazone; N-pathway = NADH-linked pathway; NADH = reduced nicotinamide adenine dinucleotide; NS-pathway = NADH and succinate-linked pathway; OXPHOS = oxidative phosphorylation; S-pathway = succinate-linked pathway; TMPD = *N,N,N',N'*-tetramethyl-*p*-phenylenediamine dihydrochloride.

addition	respiration state and pathway
malate (2 mM) + glutamate (10 mM)	LEAK state respiration, supported by N-pathway (NADH to complex I)
ADP (10 mM)	OXPHOS state respiration, supported by N-pathway (NADH to complex I)
cytochrome *c* (0.01 mM)	added to assess integrity of outer mitochondrial membrane
succinate (10 mM)	OXPHOS state respiration, supported by NS-pathway (NADH to complex I and succinate to complex II)
FCCP (titrated in 0.25 µM increments)	ET state respiration, supported by NS-pathway (NADH to complex I and succinate to complex II)
rotenone (0.5 µM)	ET state respiration, supported by S-pathway (succinate to complex II)
antimycin A (2.5 µM)	residual oxygen consumption
TMPD (0.5 mM) + ascorbate (2 mM)	ET state respiration with substrates for complex IV
sodium azide (100 mM)	inhibits complex IV, allows correction of auto-oxidation of TMPD

**Table 2.** Example substrate/uncoupler/inhibitor titration protocol (with final concentrations) to probe mitochondrial respiration supported by fatty acid oxidation and pyruvate in saponin-permeabilized placental samples, including their relative contribution to maximal respiratory capacity. ADP = adenosine diphosphate; ET = electron transfer; F-pathway = fatty acid oxidation-linked pathway; FCCP = carbonyl cyanide p-(trifluoromethoxy) phenylhydrazone; N-pathway = NADH-linked pathway; NADH = reduced nicotinamide adenine dinucleotide; NS-pathway = NADH and succinate-linked pathway; OXPHOS = oxidative phosphorylation; S-pathway = succinate-linked pathway; TMPD = *N,N,N',N'*-tetramethyl-*p*-phenylenediamine dihydrochloride.

addition	respiration state and pathway
malate (2 mM) + octanoyl-carnitine (0.2 mM)	LEAK state respiration, supported by F-pathway (fatty acid oxidation)
ADP (10 mM)	OXPHOS state respiration, supported by F-pathway (fatty acid oxidation)
cytochrome *c* (0.01 mM)	added to assess integrity of outer mitochondrial membrane
pyruvate (25 mM)	OXPHOS state respiration, supported by N-pathway (NADH to complex I)
glutamate (10 mM)	OXPHOS state respiration, supported by N-pathway (NADH to complex I)—note glutamate is added to saturate N-pathway
succinate (10 mM)	OXPHOS state respiration, supported by NS-pathway (NADH to complex I and succinate to complex II)
FCCP (titrated in 0.25 µM increments)	ET state respiration, supported by NS-pathway (NADH to complex I and succinate to complex II)
rotenone (0.5 µM)	ET state respiration, supported by S-Pathway (succinate to complex II)
antimycin A (2.5 µM)	residual oxygen consumption

Respiration rates are typically normalized to the wet mass of tissue measured before it is placed in the oxygraph chamber, but can additionally be normalized to a marker of mitochondrial content (e.g. citrate synthase enzyme activity) measured in extracts recovered from the oxygraph at the conclusion of the experiment. Alternatively, flux control ratios (FCRs) can be calculated to indicate the relative capacity of different respiratory states (for calculations see [70]). Being calculated from respiration data acquired within a single assay, FCRs are independent of tissue mass and mitochondrial content, and allow an intramitochondrial assessment of pathway capacities. An FCR might, for example, indicate the degree to which FAO can support maximal OXPHOS capacity. Finally, OXPHOS coupling efficiency [70] can be calculated from LEAK and OXPHOS rates.

High-resolution respirometry is therefore a powerful tool through which placental mitochondrial respiratory function and its control can be comprehensively assessed in an integrated, physiologically relevant system. We have not yet systematically analysed regional variation in mitochondrial respiratory capacity across the placenta, nor the generalizability of findings from a specific region, and such analysis might therefore form the basis of future work. The technique is, however, limited in that assays are carried out under conditions of saturating oxygen and substrate concentrations, and while this is effective in revealing mitochondrial respiratory capacity, it might only reflect *in vivo* respiration under maximally active conditions. Where possible, it is therefore prudent to interpret such measurements alongside markers of *in vivo* metabolism, for example we have typically found good agreement between measurements of respiratory function and metabolite levels measured in extracts of snap-frozen tissues by targeted mass spectrometry [35,49,71]. Similarly, comprehensive analysis of plasma metabolites conducted in parallel can additionally allow differences in tissue mitochondrial metabolism to be considered in the context of whole-body metabolic alterations at high altitude [82]. Moreover, while respirometry can reveal differences in mitochondrial respiratory capacity between samples, further analysis of features such as tissue mitochondrial content, ETS complex levels and supercomplex abundance might be necessary to reveal the mechanistic basis underlying such differences [83]. Finally, parallel experiments measuring mitochondrial respiration in trophoblast-like cell lines [49,62], placental fibroblasts [49], primary trophoblasts [84] or placental organoids [85] can be used to further probe mechanisms of pathology, or the influence of hypoxia, substrate availability or pharmacological therapeutics.

## 5. Conclusions

In high-altitude pregnancy, placental mitochondrial respiration is suppressed, likely playing a critical role in the partitioning of oxygen to meet fetal demands, albeit at the cost of enhanced placental glucose consumption and fetal hypoglycaemia. In Andean highlanders, high uteroplacental blood flow and enhanced maternal antioxidant capacity may protect placental OXPHOS, contributing to the relative protection of fetal growth. The capacity to comprehensively assess mitochondrial respiratory function in cryopreserved samples allows new insights into the impact of high altitude on placental bioenergetic function and signals of metabolic adaptation. This work could, in turn, bring novel mechanistic insights that might translate to a better understanding of pregnancies complicated by oxidative stress and/or reduced uteroplacental blood flow at sea level.

## Data Availability

This article has no additional data.
